# Bisphenols A and F, but not S, induce apoptosis in bovine granulosa cells *via* the intrinsic mitochondrial pathway

**DOI:** 10.3389/fendo.2022.1028438

**Published:** 2022-10-28

**Authors:** Emilia Kourmaeva, Reem Sabry, Laura A. Favetta

**Affiliations:** Reproductive Health and Biotechnology Laboratory, Department of Biomedical Science, Ontario Veterinary College, University of Guelph, Guelph, ON, Canada

**Keywords:** endocrine disrupting compound, BPA, BPF, apoptosis, oocyte competence, fertility, BPS

## Abstract

With the gradual decline in global fertility rates, there is a need to identify potential contributing factors, their mechanisms of actions and investigate possible solutions to reverse the trend. Endocrine disrupting compounds (EDCs), such as bisphenol A (BPA), are environmental toxicants that are known to negatively impact reproductive functions. As such, the use of BPA in the manufacturing industry has slowly been replaced by analogs, including bisphenol S (BPS) and bisphenol F (BPF), despite limited knowledge available regarding their impact on health and their safety. The following study investigates the effects of BPA, BPS and BPF at a concentration of 0.5 μg/mL and 50 μg/mL on bovine granulosa cell apoptosis, with the ultimate goal of determining how they may impact oocyte competence and, thus, overall fertility. The underlying hypothesis is that bisphenols disrupt the granulosa cell environment surrounding the oocyte inducing excessive apoptosis *via* the intrinsic mitochondrial pathway. To test this hypothesis, apoptosis was measured following a time- and dose-dependent exposure to all three bisphenols by flowcytometry paired with annexin V/PI staining as well as by quantification of key genes belonging to the intrinsic apoptotic pathway both at the mRNA and protein levels. The results of this study report that BPA and BPF reduce cell viability through reduced cell counts and increased apoptosis. This increase is due, in part, to the induction of apoptotic genes of the intrinsic pathway of apoptosis. Additionally, this study also suggests that BPS may not act on the intrinsic mitochondrial apoptotic pathway in bovine granulosa cells. Overall, this study allows us to establish potential apoptotic pathways activated by bisphenols as well as compare the relative apoptotic activities of BPA to its most widespread analogs.

## Introduction

An alarming trend that can be observed in several countries is the decline in total fertility rates (TFR), referring to the number of children that a woman would have throughout her reproductive life if she experienced fertility rates that are age-specific during a given year (1). In 2000, the TFR was 2.72 and dropped to 2.31 by 2019, with the United Nations projecting the TFR to continue decreasing (2, 3). Although there are multiple factors that play a role, including lifestyle choices *in primis*, one noteworthy contributor to this decline is an increase in both female and male infertility rates (4). Increased exposure to man-made environmental pollutants and toxicants, such as endocrine disrupting compounds (EDCs), is known to have negative impacts on reproductive health, thus is suspected to contribute to the increase in infertility rates (5).

With more than 6 billion pounds being produced each year and 10.6 million metric tons predicted to be produced in 2022, one of the most widely used EDCs is bisphenol A (BPA) (6–8). BPA is an industrial chemical used in manufacturing various plastics and resins that can be found in numerous consumer products such as plastic food and beverage containers and in the lining of food cans, to name a few (6–8). BPA acts as a xenoestrogen that is capable of binding to various receptors such as estrogen receptor alpha and beta (ERα and ERβ), eliciting endocrine disrupting effects (9). BPA has been associated with female reproductive damage *via* delayed oocyte maturation, diminished ovarian reserve, increases in follicular atresia, increases in oxidative stress, changes in gene expression, and overall reduction in developmental capabilities to produce a healthy offspring (10–14). Due to the negative health outcomes associated with BPA, many countries have taken steps toward replacing BPA with alternatives, including its structural analogs, bisphenol S (BPS) and bisphenol F (BPF), despite limited knowledge available regarding their effects on health and a great chance that they would also share comparable physiological and toxicological effects considering their structural similarities (15). BPS and BPF are now used in the manufacturing industry to replace BPA in plastics, lining of aluminum cans, and various products such as thermal paper, increasing their prevalence in the environment (16). With the absence of regulations for their use, both analogs are now widely detectable in the population and concerning consequences following their exposure have been noted (16).

Successful female reproduction relies on oocyte competence. In order for an oocyte to maintain its competence, it requires the proper microenvironment which is created by the surrounding granulosa cells and follicular fluid (17). Granulosa cells support oocyte development, and maturation, while also playing a protective role, by secreting factors that protect the oocyte from reactive oxygen species and oxidative damage (18, 19). Granulosa cells that surround the oocyte are essential in creating the proper microenvironment needed for the oocyte to mature properly and maintain competence. For an oocyte to be considered competent, it must have the ability to resume meiosis, be fertilized, and sustain development to the blastocyst stage (20) Granulosa cells maintain a bi-directional communication with the oocyte whereby, through gap junctions, they supply the oocyte with nutrients, metabolites, and molecular signals (21). Zhou et al. found that oocytes matured without cumulus cells (the granulosa cells in closest proximity to the oocyte) displayed a significant decrease in nuclear maturation compared to those matured with cumulus cells, and this was not reversed with co-culturing (22). Their results suggest that for efficient oocyte maturation to occur, granulosa cells and their intact gap junctions with the oocyte are required (23). Further, when looking at insemination, the percentage of 2-cell-stage embryos produced was decreased in the oocytes cultured without granulosa cells during fertilization (23). These cells are susceptible to the effects of toxins in the follicular fluid, such as bisphenols, which can impact the oocytes development and maturation, thus hindering oocyte competence (24). Due to their importance for overall fertility, a reduction in granulosa cell viability could result in decreased success of reproduction in females. Therefore, granulosa cells are often studied as reliable markers for oocyte developmental potential and present excellent models for toxicity studies in reproduction (25).

While growing literature is available regarding the effects of bisphenols, a gap in knowledge surrounding the exact mechanisms of action of BPA and its alternatives on the viability and apoptosis of granulosa cells, and whether these analogs are safer than their predecessor, still exists. BPA’s potential to induce programmed cell death, otherwise referred to as apoptosis, has previously been reported in a variety of cells, including human bronchial epithelial cells, rhesus monkey embryo epithelial cells and human granulosa KGN cells (26–28). BPA was also found to induce apoptosis in blastocysts from treated bovine oocytes (11). The intrinsic pathway of apoptosis is a process of cellular death that occurs as a result of intracellular stressors, such as exposure to toxins, on DNA stability and mitochondrial membrane potential (29). Previous studies treating oocytes with BPA found increased apoptosis in the produced blastocysts through TUNEL staining indicating increased DNA fragmentation (12, 18) Furthermore, multiple studies have reported BPA induced dysregulation of intrinsic apoptotic genes such as BAX, BCL-2, Caspases, and even heat shock proteins (30). These genes are interconnected in a pathway that regulates mitochondrial membrane potential and functions to balance the presence of anti- and pro- apoptotic molecules around the mitochondria based on the needs of the cell (30). As such, it is suspected that bisphenols are capable of exerting their mechanisms of action through the mitochondria to induce granulosa cell apoptosis. Further, due to their role in providing growth and survival factors, increased apoptosis of granulosa cells is thought to negatively impact the oocyte, and ultimately fertility (31). While there are fewer studies available regarding BPS and BPF, some have shown both analogs to have apoptotic potential (32, 33), however the mechanism through which they induce apoptosis is not clearly established.

In the present study, bovine granulosa cells were used as an experimental model for humans as it has been established to be an excellent translational model to study female reproductive toxicology in humans and in livestock. Both bovine and humans share many common characteristics when it to comes to reproductive biology including oocyte diameter and morphology, ovarian physiology and stages of embryogenesis, to name a few (34). As such, the research presented in this study utilizes bovine granulosa cells to determine the effects of BPA and its analogs on the surrounding environment of the oocyte, which determines its developmental potential. This study further aims to expand the knowledge available on the mechanisms of action of bisphenols, with the ultimate goal of establishing a relationship between bisphenols and female infertility.

## Materials and methods

### Granulosa cells collection & *in-vitro* granulosa cell culture

The bovine (Bos Taurus) granulosa cells used in this study were previously isolated as described by Sabry and colleagues (35). Briefly, bovine ovaries were collected from local abattoirs (Cargill Meat Solutions, Guelph, Ontario, Canada & Highland Packers, Stoney Creek, Ontario, Canada) and transported to the laboratory under temperature-controlled conditions between 34-36° C. Cumulus Oocyte Complexes (COCs) were then aspirated from antral follicles into collection media.

The COCs were mechanically denuded and granulosa cells were washed in media including 1X Dulbecco’s Modified Eagle Medium (DMEM) (Gibco), penicillin/streptomycin (1%) and glutamine (2mM) (Sigma-Aldrich), resuspended in DMEM supplemented with 20% fetal bovine serum (FBS) (Gibco), plated in a T75 flask (Corning) and incubated at 38.5°C in 5% CO_2_ until confluent, with media replacement every 48 hours. Once confluent, passage 2 cells were trypsinized (Sigma Aldrich) and split into 6-well plates at a seeding density of 1 x 10^5^ cells/mL in DMEM supplemented with 10% FBS. 24 hours after, cells were serum restricted in Opti-MEM Reduced Serum Medium (Thermo Fisher) for 24 hours, and then randomly assigned to one of the following treatment groups: Opti-MEM (Control), 0.1% ethanol in Opti-MEM (Vehicle), 5 μg/mL estradiol in Opti-MEM (Estradiol), 0.5 μg/mL of BPA, BPS or BPF in Opti-MEM (“Low dose”) and 50 μg/mL of BPA, BPS or BPF in Opti-MEM (“High dose”). The “high dose” groups were exposed to the current LOAEL (50 μg/mL) dose that is established for BPA, while the dose that is 100x lower than the LOAEL (0.5 μg/mL) is denoted as the “low dose” (10). When looking at the effects of an exogenous treatment on cellular parameters including gene expression, it is important to ensure that the cells are synched to the same cell cycle phase, also known as *in vitro* synchronization. This ensures that the differences seen among groups are due to the treatment and not due to extrinsic factors in cell cycle stage. Serum deprivation is a commonly used method to synchronize the cell cycle (36)

Following treatment for either 12 or 48 hours, live cells were imaged using the EVOS XL core imaging system (AMEX1000) and then either snap frozen in liquid nitrogen or used immediately for subsequent experiments. These doses and times were chosen based on a dose- and time- dependent cell viability assay previously performed by our group (14).

### Live cell counts

To obtain live cell counts, a trypan blue exclusion assay was performed using the Bio-Rad TC10 Automated Cell Counter. Following treatment, cells and trypan blue (Gibco) were pipetted onto each side of a counting slide and placed into the automated cell counter. After performing two counts on each replicate, the average was taken. This was executed on a minimum of three biological replicates.

### Annexin V-FITC and PI apoptosis detection

To quantify the levels of apoptosis in treated granulosa cells, the Annexin V-FITC Apoptosis Staining/Detection Kit was used (Abcam – ab14085), as previously described with minor modifications from Dufour et al., {*Submitted}*. After treatments, the spent media was placed into centrifuge tubes to account for dead cells. Cells were then detached using TrypLE Express (Gibco) as a gentler substitute to trypsin and added to the tube with the spent media. Cells were spun down at 6° C for 5 minutes at 5000 xg and the supernatant was removed. Cells were resuspended in binding buffer supplemented with 10% FBS and incubated for 5 minutes. 10 μL of the Annexin V-FITC reagent was added to each tube and incubated for 30 minutes. 5 μL of PI was added and incubated for 5 minutes. During the staining process, three control tubes were included; one no stain, one containing the Annexin V-FITC stain only, excluding PI, and another containing the PI stain only, excluding the Annexin V-FITC stain. Cells were then pipetted through a 40 μL strainer (VWR) into Flow tubes (Fisher Brand) and placed on ice. The tubes were then placed in the BD Accuri C6 Flow Cytometer. Cell count limit was set to 50,000, and the fluidics rate to slow. Results were analyzed using the FlowJo software for a minimum of three biological replicates.

### RNA extraction and cDNA synthesis

RNA was extracted from frozen granulosa cells using the RNeasy Plus Micro Kit (Qiagen) following the manufacturers protocol and as previously described by Sabry et al. (37)). Briefly, Buffer RLT Plus was added to each sample. The lysate was then transferred to a gDNA Eliminator Spin Column and centrifuged. 70% ethanol was added to the flow-through and transferred to an RNEasy MinElute Spin Column. The column was then washed with buffers and dried before eluting the RNA in RNase-free water. The concentrations of RNA were measured using a Nanodrop 2000c (ThermoFisher Scientific).

mRNA was reverse transcribed (RT) into cDNA using the QuantaBio qScript cDNA Supermix (VWR). Briefly, 4μL of the qScript cDNA Supermix was added to 0.5μg of RNA and reverse transcribed. Two controls were included: a no template control (NTC) that excluded RNA, and a no reverse transcriptase (NRT) that excluded the reverse transcriptase enzyme. The T100 Thermal Cycler (BioRad) was used to reverse transcribe the mRNA at the following settings: 5 minutes at 25° C, 30 minutes at 42° C and 5 minutes at 85° C. The samples were then stored at -20°C for qPCR analysis.

### Quantitative polymerase chain reaction

To determine and quantify the mRNA expression of three pro-apoptotic (BAX, BAD and caspase-9) and two antiapoptotic genes (HSP70 and BCL-2), the CFX96 Touch Real-Time PCR Detection System from Bio-Rad was used. Using the SsoFast EvaGreen Supermix (BioRad), each mRNA target was amplified as described by Saleh and colleagues (12) The primers used for qPCR analysis can be found in **Table 1** along with their corresponding efficiencies, determined using standard curves. Changes in mRNA expression were calculated using the efficiency-corrected method (ΔΔCt) with tyrosine 3-monooxygenase/tryptophan 5-monooxygenase activation protein zeta (YWHAZ) and peptidylprolyl isomerase (PPIA) used as reference genes. Both genes were used as reference genes as they were previously established to be unaffected with stable expression among the studies’ treatment groups (14). Further, a calibrator consisting of cDNA from control granulosa cells was used to account for inter-run variability. A minimum of three biological replicates and three technical triplicates were quantified for each primer.

**Table 1:** Primer sequences used in qPCR.

Gene Symbol	Gene Name	Accession #	Primer Sequence Sets (5’- 3’)	Efficiency (%)	Source
BAX	BCl-2 Associated X- Protein	NM_173894.1	F:TTTGCTTCAGGGTTTCATCCA R:CCGATGCGCTTCAGACACT	101.6	(38)
BAD	BCl-2 Associated Agonist of Cell Death	NM_001035459.1	F:CTTTTCTGCAGGCCTTATGC R:GGTAAGGGCGGAAAAACTTC	100.7	(39)
Casp-9	Caspase - 9	NM_001205504.1	F:AGCAAATGGTCCAGGCTTTG R:ATTCTCTCGACGGACACAGG	102.8	(40)
HSP70	Heat Shock Protein 70	NM174550	F:AACAAGATCACCATCACCAACG R:TCCTTCTCCGCCAACGTGTTG	101.1	(41)
BCL-2	B-Cell Lymphoma-2	NM_001077486.2	F:CCCGCTGCGACAGTTATA R:AACAAGGCTACGATAACCGAGAGA	100.7	(42)
YWHAZ	Tyrosine 3-Monooxygenase/Tryptophan 5-Monooxygenase Activation Protein Zeta	NM_174814.2	F:GCATCCCACAGACTATTTCC R:GCAAAGACAATGACAGACCA	99.7	(43)
PPIA	Peptidylprolyl Isomerase A	NM_178320.2	F:TCTTGTCCATGGCAAATGCTG R:TTTCACCTTGCCAAAGTACCAC	101.6	(43)

### Caspase-9 activity assay

To quantify the activity of caspase-9, the Abcam Caspase 9 Assay Kit (Abcam) was used. Cells were cultured as described above with the exception of using a 96-well plate and a seeding density of 5x10^4^ cells/mL per group. Furthermore, all measurements were performed in duplicates for each biological replicate. Lastly, each incubation period (with and without serum) was 12 hours, with the treatment exposures remaining the same at 12 or 48 hours. Cells were serum starved in Opti-MEM and treated with the same concentrations as defined previously. After treatment, 50 μL of the provided cell lysis buffer was added to each well and left to incubate for 30 minutes on ice. 50 μL of Reaction Buffer containing DTT was added to equal concentrations of protein per sample followed by 5 μL of the 1 mM LEHD AFC substrate and left to incubate at 37°C for 2 hours. The samples were then read using the SpectraMAX i3 plate reader. Blank wells were used to normalize readings against background fluorescence and the fold-increase was calculated relative to the control sample. This was performed on a minimum of three biological replicates.

### Western blotting

BAX and BCL-2 proteins were quantified by Western blotting. Cells were lysed in radioimmunoprecipitation assay (RIPA) buffer with protease inhibitors (Biotool). Samples were subjected to 4x freeze/thaw cycles in liquid nitrogen, sonicated for 30 minutes in ice water, and then centrifuged at 12,000 xg at 4°C for 10 minutes. To determine protein concentrations, a Bradford Assay was performed as described in the manufacturers protocol (BioRad) and 15% polyacrylamide gels were prepared using BioRad standard gel recipes.

20 μg of protein for each sample were dissolved in equal volumes of 3x Reducing Buffer plus 5% β- mercaptoethanol (Sigma Aldrich), denatured at 90°C and loaded onto a 15% polyacrylamide gel in the XCell SureLock Mini-Cell Electrophoresis System (Invitrogen). Gels were run at 125 V for 2 hours and proteins were transferred onto a nitrocellulose membrane (Bio-Rad) for 1 hour at 20V using the Invitrogen Wet Transfer Western blot apparatus (Invitrogen) filled with 1x Towbins buffer. Membranes were washed in 1x Tris Buffered Saline and 0.1% Tween (TBST) (FisherBrand) and then blocked for 1 hour in the 5% skim milk or 5% BSA in TBST for BAX (Proteintech; 50599-2-IG) and BCL-2 (Proteintech; 12789-1-AP), respectively. Primary antibodies of BAX or BCL-2 were added for 1.5 hours at room temperature. BAX primary antibody was diluted to a concentration of 1:1000 in 5% skim milk in TBST, while BCL-2 was diluted to a concentration of 1:1000 in 5% BSA in TBST. After washes, the anti-rabbit IgG HRP-linked secondary antibody (Cell Signalling Technology; 70735) diluted at a concentration of 1:3000 was added for 1 hour at room temperature. Blots were incubated in Clarity Western ECL Blotting Substrate (Bio-Rad 170-5060) and protein bands were detected using the Bio-Rad ChemiDoc XRS + Imaging System. β-actin (Cell Signalling Technology, 4967) at a dilution of 1:2000 in 5% BSA in TBST was used as loading control with a secondary anti-mouse IgG HRP-Linked antibody (Cell Signalling Technology, 7076) at a dilution of 1:5000 in 5% skim milk in TBST for 1 hour. Densitometric analysis was performed using the Image Lab software from Bio-Rad and the protein levels were expressed as a ratio to β-actin in each sample. Lastly, the ratio of BAX/BCL-2 protein in the treated samples was calculated as the proportion of normalized BAX to normalized BCL-2 within the same biological samples.

### Statistical analysis

GraphPad Prism 6 statistical software was used to analyze statistical difference. To determine normality, each data set was tested using the Shapiro-Wilk test. Data sets that were determined to be normally distributed were analyzed using the one-way analysis of variance (ANOVA), while non-normal data sets were analyzed using the Kruskal-Wallis test. Three biological replicates were used in each experiment, at a minimum, while statistical significance was determined at a two-tailed p-value <0.05. If the p-value was statistically significant, data sets were subjected to Tukey’s *post-hoc* test, while non-significant data underwent Dunn’s multiple comparison test, comparing differences between each treatment group. Data shown represent the mean +/- standard error of the mean (SEM).

## Results

### Granulosa cell morphology

**Figure 1:**
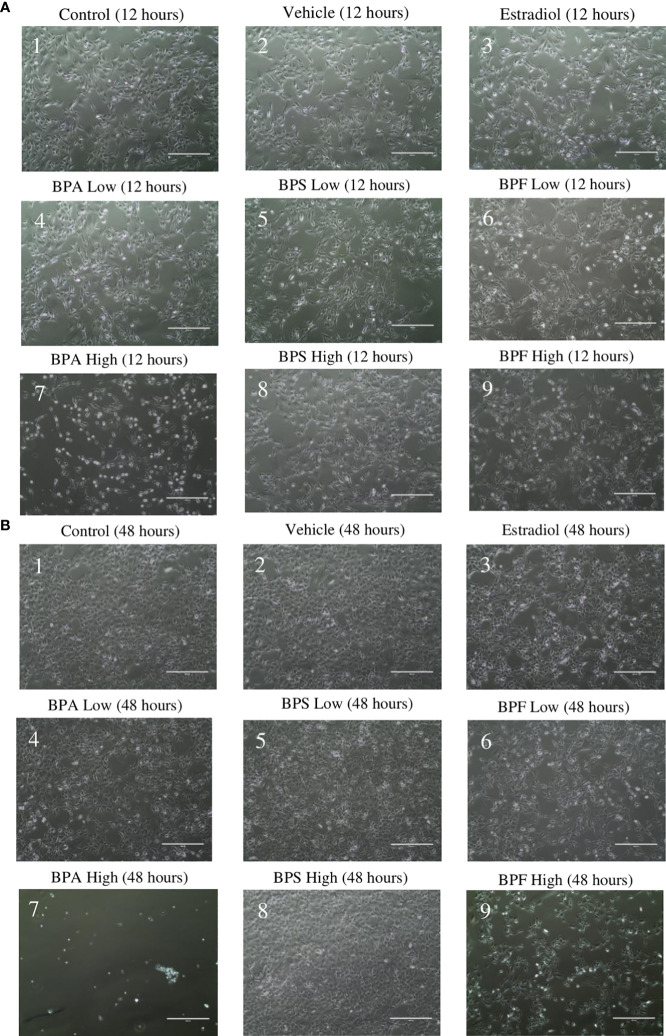
Granulosa cell morphology. After 12 hours **(A)** and 48 hours **(B)** of treatment with environmentally significant doses of bisphenols (A, S, F). Images were taken using the ThermoScientific EVOS digital inverted microscope at 10x magnification. Scale bar=1000μm.

Following treatment, cell morphology was observed under a digital inverted microscope. At the 12 hour timepoint (**Figure 1A**), cells in the control, vehicle, estradiol, BPA and BPS low dose groups appeared similar in terms of confluency and shape (**Figures 1A1–A5**). Cells treated with BPF at the low dose (**Figure 1A6**) appeared less confluent than the control group, with more cells that detached from the flask and apparent shrinkage in cell shapes. This was also observed in cells treated with BPA and BPF at the high dose (**Figures 1A7, A9**). Cells treated with BPS at the high dose appeared similar in terms of confluency to the control and vehicle, with little to no visible morphological changes (**Figure 1A8**). At the 48 hour timepoint (**Figure 1B**), cells treated with estradiol, BPA and BPF at the low dose were less confluent compared to cells in the control and vehicle. BPA at the high dose (**Figure 1B7**) appeared lethal to cells, with little to no adherent cells left in the well. The cells treated with BPF at the high dose (**Figure 1B9**) were markedly less confluent to the control and vehicle at this timepoint. This effect was not present for BPS at either dose.

### Live cell counts

**Figure 2:**
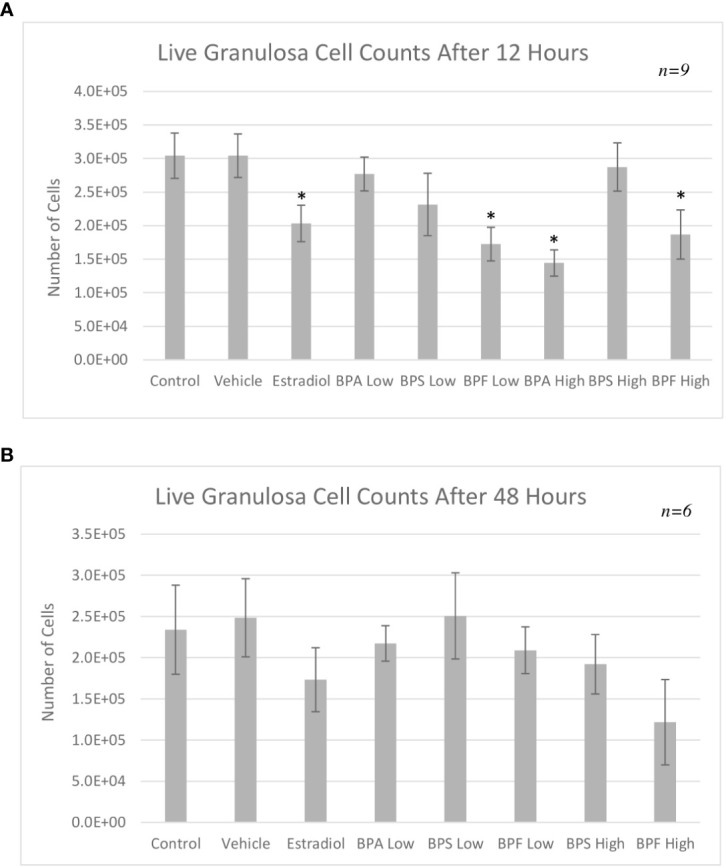
Live granulosa cell counts using the TC10 Automated Cell Counter. Cell counts were obtained following treatment with low dose (0.5 μg/mL) and high dose (50 μg/mL) BPA, BPS and BPF. **(A)** depicts the 12-hour timepoint and **(B)** displays the 48-hour timepoint. *p<0.05 compared to controls was considered statistically significant. Error bars represent +/- SEM.

Following treatment, live granulosa cell counts were obtained using trypan blue exclusion as a method to quantify cell viability. The cell count for each biological replicate was averaged and statistically compared (**Figures 2A, B**). After 12 hours of treatment, the control and vehicle groups had similar cell counts. There were significantly less cells in the estradiol treated group (p<0.05). Similar to what was observed after imaging, cells treated with BPA and BPS at the low dose and BPS at the high dose were not significantly different compared to the control and vehicle in terms of count. Furthermore, there were significantly less cells in the groups treated with BPF at the low dose (p=0.0188) and BPA and BPF at the high dose (p=0.0215 and p=0.0343, respectively) compared to the control and vehicle (**Figure 2A**).

After 48 hours of treatment, there was no statistically significant difference among the treatment groups (**Figure 2B**). Both the control and vehicle groups had similar cell counts with estradiol displaying a slight decrease, similar to the results seen at the 12 hour timepoint. BPA at the high dose after 48 hours was found to be lethal to cells. Since the automated cell counter is not able to count under 5 x 10^4^ cells/mL, and since treatment with this high-dose BPA was below this value, this group was not included in the analysis or subsequent experimentation. Although not statistically significant, there was a consistent decrease in cell counts in the BPF high dose treated cells, which corresponds to changes in confluency seen in the images of **Figure 1**.

### Annexin V-FITC and PI apoptosis detection

**Figure 3:**
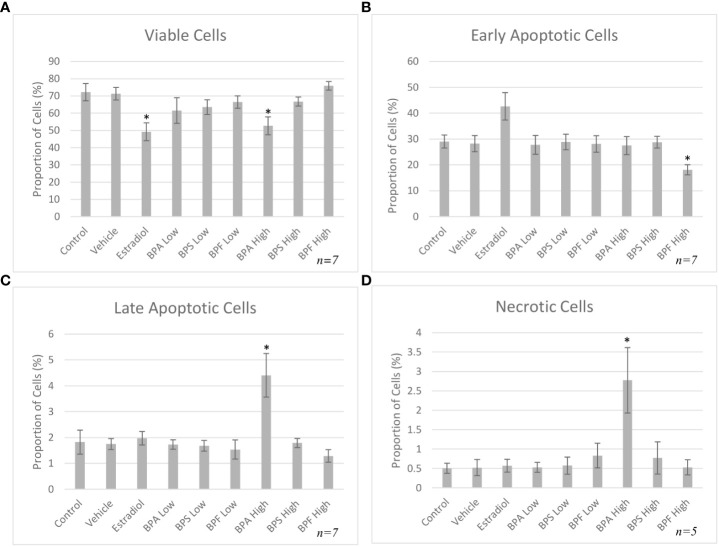
Apoptosis rates of bovine granulosa cells at 12 hours. This was detected by Annexin V-FITC/PI staining and flow cytometry. Columns represent the proportion **(A)** viable cells, **(B)** early apoptotic cells, **(C)** late apoptotic cells and **(D)** necrotic cells following treatment with BPA, BPS and BPF at two different concentrations for 12 hours. *p<0.05 compared to controls was considered as statistically significant. Error bars represent +/- SEM.

Flow cytometry using Annexin V-FITC/PI staining was performed to distinguish between the proportion of viable, early/late apoptotic and necrotic cells following treatment. As shown in **Figure 3A**, the percentage of viable cells (Annexin V-/PI-) after 12 hours of treatment significantly decreased in the estradiol group and the BPA high group (P=0.0472 and 0.0292, respectively). As seen in **Figure 3B**, there was a noticeable decrease in cells undergoing early apoptosis (Annexin V+/PI-negative) in the BPF at the high dose (p=0.032). Among all the treatment groups, only cells in the BPA high dose group underwent a significant proportion of late apoptosis (p<0.05) (Annexin V+/PI+) as well as necrosis (p=0.0486) (Annexin V-/PI+) (**Figures 3C, D**).

**Figure 4:**
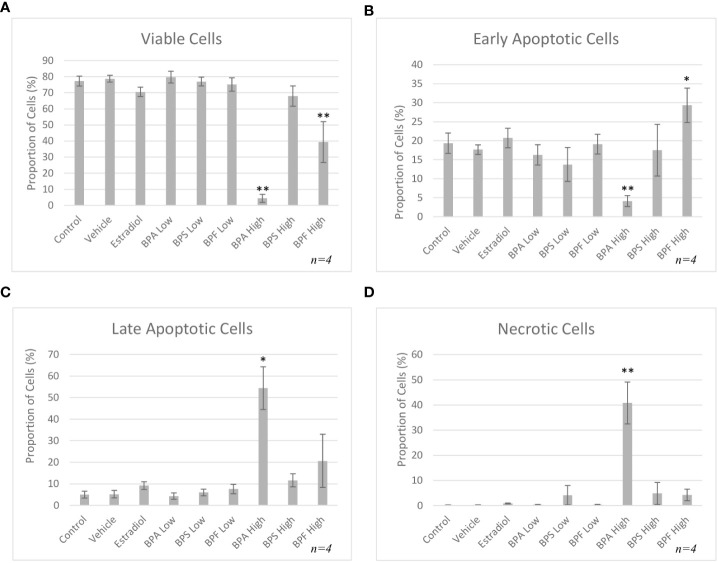
Apoptosis rates of bovine granulosa cells at 48 hours. This was detected by Annexin V-FITC/PI staining and flow cytometry. Columns represent the proportion **(A)** viable cells, **(B)** early apoptotic cells, **(C)** late apoptotic cells and **(D)** necrotic cells following treatment with BPA, BPS and BPF at two different concentrations for 48 hours. *p<0.05 and **p<0.01 compared to controls was considered as statistically significant. Error bars represent +/- SEM.

Following 48 hours of treatment, a significant decrease in viability was detected in the BPA and BPF high dose groups, p= 0.003 and p=0.009 respectively, with a decrease of 73% and 38% in each group compared to control (**Figure 4A**). This was confirmed by a significant increase in cells undergoing early apoptosis in the BPF high dose group (p<0.05) (**Figure 4B**). Cells in the BPA high dose group experienced a significant decrease in the proportion of early apoptosis (p=0.003) coupled with a significant increase in late apoptosis (p=0.03) (**Figure 4C**), corresponding to the decrease in viability previously observed. A significant proportion of cells were undergoing necrosis in the BPA high dose group (p=0.007) (**Figure 4D**), supporting the exclusion of this treatment group from subsequent experimentation due to lack of starting material to obtain enough RNA and protein for quantification.

### mRNA expression

**Figure 5:**
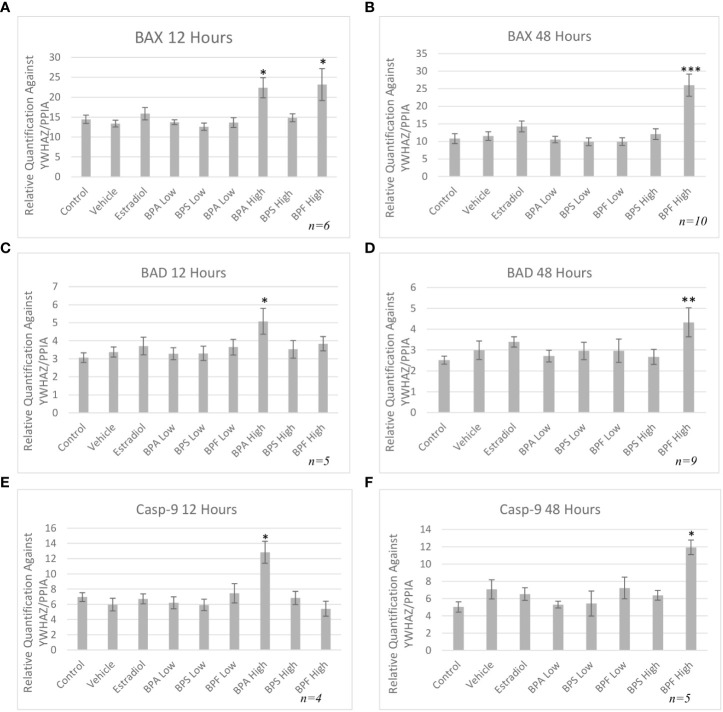
Pro-apoptotic mRNA expression after treatment with bisphenols. BAX 12 **(A)** and 48 **(B)** hours, BAD 12 **(C)** and 48 **(D)** hours, and Casp-9 12 **(E)** and 48 hours **(F)** mRNA in granulosa cells after treatment at a low (0.5 μg/mL) and high (50 μg/mL) dose of BPA, BPS, and BPF. Quantification relative to reference genes YWHAZ and PPIA. *p < 0.05,**p<0.01, and ***p<0.001 compared to controls was considered as statistically significant. Error bars represent +/- SEM.

**Figure 6:**
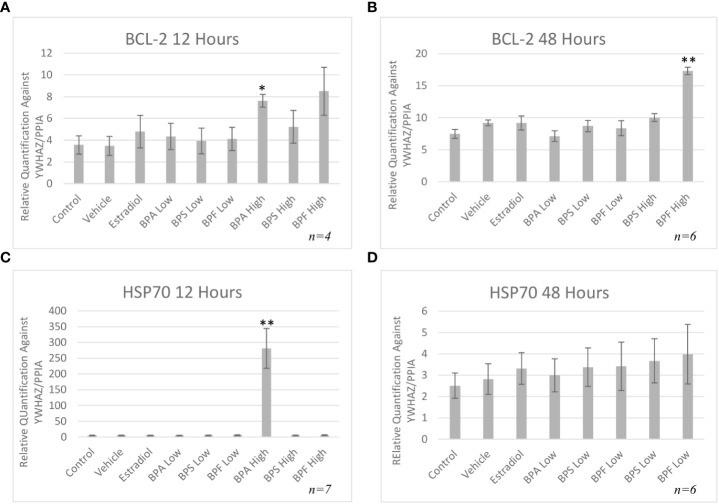
Anti-apoptotic mRNA expression after treatment with bisphenols. BCl-2 12 **(A)** and 48 **(B)** hours, and HSP70 12 **(C)** and 48 **(D)** hours in granulosa cells after treatment at a low (0.5 μg/mL) and high (50 μg/mL) dose of BPA, BPS, and BPF. Quantification relative to reference genes YWHAZ and PPIA. *p < 0.05 and **p < 0.01 compared to controls was considered as statistically significant. Error bars represent +/- SEM.

mRNA levels of three pro-apoptotic (BAX, BAD and caspase-9) and two anti-apoptotic (BCL-2 and HSP70) genes were quantified by qPCR relative to two reference genes (YWHAZ and PPIA) after both 12 and 48 hours of treatment. As seen in **Figure 5A**, mRNA expression of BAX was significantly increased in the BPA and BPF high dose groups following 12 hours of treatment (p=0.046 and p=0.032, respectively). This trend continued following 48 hours treatment as seen in **Figure 5B**, where BAX expression was significantly upregulated in the BPF high dose group (p=0.0002). BPA at the high dose was lethal to cells at the 48 hour timepoint, therefore relative mRNA expression with this treatment was excluded at this timepoint. BAD and Caspase-9 expression were also significantly increased in the BPA high dose group following 12 hours and the BPF high dose group following 48 hours of treatment (p=0.049/0.049 and p=0.0039/0.0092, respectively) (**Figures 5C-F**). mRNA expression was also significantly increased for BCL-2 in the BPA high dose group after 12 hours and in the BPF high dose group after 48 hours (p=0.015 and p=0.0012) (**Figures 6A, B**). For HSP70, a significant increase in expression was detected at the 12 hour timepoint for BPA at the high dose (p=0.006) (**Figure 6C**). After 48 hours, no significant changes in mRNA expression were detected among treatment groups (**Figure 6D**).

### Caspase-9 activity

**Figure 7:**
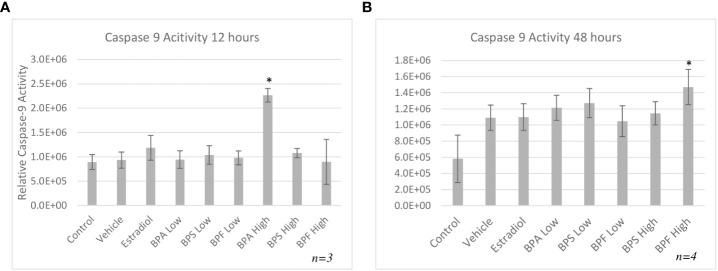
Activity of caspase-9 after 12 or 48 hours of treatment. Cells were treated with low dose (0.5 μg/mL) and high dose (50 μg/mL) BPA, BPS and BPF. **(A)** represents activity following 12 hours and **(B)** represents activity following 48 hours. *p<0.05 compared to controls was considered as statistically significant. Error bars represent +/- SEM.

The bioactivity of caspase-9 was measured using a fluorometric assay kit whereby samples were incubated with the caspase-9 substrate LEHD-AFC following treatment. **Figure 7A** depicts the caspase-9 activity after 12 hours of treatment, which was significantly increased in the BPA high dose group (p=0.013). After 48 hours (**Figure 7B**), caspase-9 activity was significantly increased (p=0.05) in the BPF high dose group.

### Protein expression

**Figure 8:**
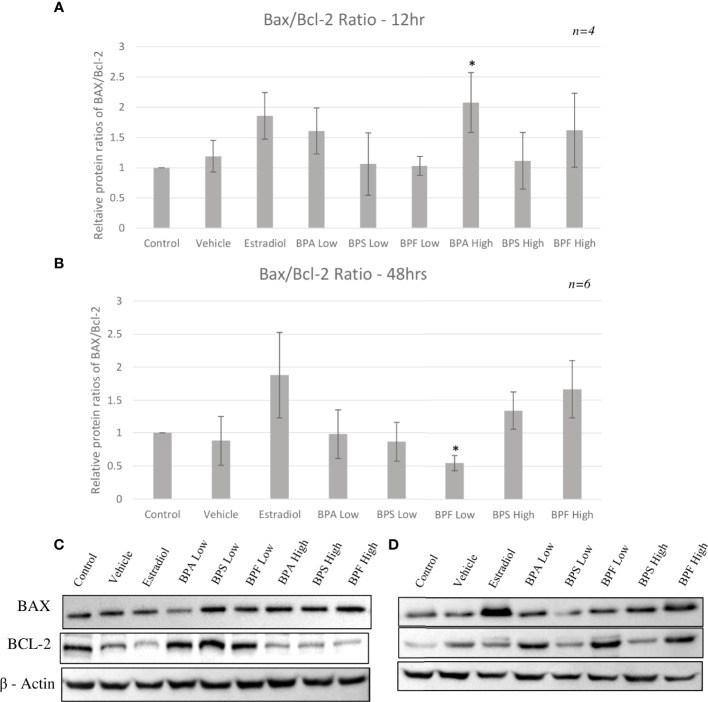
Protein ratios of pro-apoptotic BAX and anti-apoptotic BCL-2. Expression was normalized relative to β -actin and was quantified after bisphenol treatment with BPA, BPS and BPF at 0.5 μg/ml and 50 μg/mL. Ratios were then calculated to obtain the proportion of BAX to Bcl-2 in the same biological samples after **(A)** 12 hours and **(B)** 48 hours. Blots represent BAX, BCL-2, and B-actin at **(C)** 12 hours and **(D)** 48 hours. *p<0.05 compared to controls was considered as statistically significant. Error bars represent +/- SEM.

Protein expression of pro-apoptotic BAX and anti-apoptotic BCL-2 was quantified by Western blotting relative to the loading control β - actin following 12 or 48 hours of treatment with BPA, BPS and BPF at 0.5 μg/ml and 50 μg/ml. **Figures 8A, C** shows that the majority of treatments did not affect the BAX/BCL-2 ratio compared to control at 12 hours, however, there was a significant increase in the BPA high dose group in favor of BAX (p=0.0231). **Figure 8B** displays this ratio after 48 hours of treatment showing a significant decrease of BAX/BCL-2 in favor of BCL-2 in the BPF low group (p=0.0209). There was a notable trend towards increased ratio levels in favor of BAX in the Estradiol and BPF high dose groups.

## Discussion

With the ongoing rise in infertility and the global fertility rates expected to continue decreasing, investigating potential causes that could lead to long term fertility improvements is of high interest. Among several reasons behind infertility, exposure to environmental toxicants, such as bisphenols, was shown to be a strong contributor to the decline in fertility (44). BPA’s endocrine disrupting potential and adverse reproductive effects are well-established and have led to restrictions for its use and the introduction of alternatives analogs, such as BPS and BPF. As such, products containing the analogs are labeled as “BPA free” giving consumers the impression that they are safer, however, their adverse effects and potential impacts on fertility are not well established. Structurally, these analogs resemble BPA and were shown to display the same endocrine disrupting potential and effects *via* nuclear receptors, as BPA (45). More recent studies also reported their impacts on health (45). Thus, the question of whether these alternatives are indeed safer still remains, strengthening the importance of our study. Here we aimed to establish whether these analogs have a lower ability of reducing viability and inducing apoptosis, while also aiming to understand mechanisms of BPA-induced apoptosis. Granulosa cells play an important role in oocyte competency, thus, if bisphenols impact these cells, they impact oocyte maturation capability and overall fertility.

This study observed changes in cell morphology and confluency after bisphenol treatment. Like BPA, BPF-treated groups were less confluent and smaller in size, rounder, visibly thinner, and less polygonal (46). Harnett et al. found that BPA and another analog, BPAF, caused growth and morphological changes in human and rat stem cells (46). In both species, cells appeared to have shrinkage, while many also lost their attachment to the culture plate, as also observed in our study (46). Further, Maćczak et al. showed that BPA and BPF induced morphological changes in human erythrocytes, signifying that these compounds were cytotoxic to cells, while BPS did not cause any changes in morphology (22). This suggests BPF behaves more like BPA than BPS in bovine granulosa cells.

Cell counts provided a quantitative approach in determining the effects of bisphenols on cell viability. Live cell counts were significantly decreased in the estradiol group after 12 hours (**Figure 2A**). While this was an unexpected result, Li and colleagues suggest that 17-β-estradiol can induce apoptosis through the mitochondrial pathway (20). This is triggered by binding to phosphodiesterase 3A, which recruits the protein SLFN12 that binds to ribosomes, leading to blocked protein translation in the endoplasmic reticulum, and the downregulation of BCL-2, triggering the process of apoptosis (20). Aside from estradiol, there was a significant decrease in cell viability in the BPF low dose and BPA and BPF high dose groups supporting BPFs similarity to BPA. These findings are similar to those of Qi and colleagues who suggested that as concentrations of BPA increase, human granulosa cell viability decreases (21). Although there are no studies that have explored BPF effects on granulosa cell viability, studies in human breast cancer cells, peripheral blood mononuclear cells and placenta cells showed that exposure to BPF also led to decreased cell viability (33, 47, 48). Structures of BPA, BPF, and estrogen are similar allowing these xenoestrogens to bind to and activate ERs, disrupting estrogen signaling leading to downstream consequences, such as apoptosis, with changes in morphology and decreased cell viability (49).

In order to further measure the effects of bisphenols, Annexin V/PI staining followed by flow cytometry was carried out. After 12 hours, a significant decrease in viable cells in the estradiol-treated group and in the BPA high dose group compared to control was detected. Overall, the BPA high dose caused a significant proportion of late apoptosis and necrosis, which is expected for BPA and supported in literature (50, 51). Using a similar method of flow cytometry, Liang et al. subjected mouse preantral follicular cells to BPA at various concentrations to determine effects on viability (52). Lower doses of BPA induced less apoptosis, while greater doses as high as 200 μM/L, which is similar to the high dose in the present study, greatly increased apoptosis through reduced mitochondrial membrane potential (52). As previously mentioned, BPA and BPF have been shown to induce apoptosis in a variety of cells, including embryo epithelial cells, KGN cells, blastocysts and mouse oocytes (11, 27, 28, 53). Our results suggest that BPA and BPF are inducing apoptosis, which would in turn decrease the important nutrients, metabolites and signals provided to an oocyte. This could lead to decreased maturation rates and lower number of 2-cell-stage embryos, thus impacting fertility rates.

To investigate the apoptotic pathway activated by bisphenols, the mRNA expression of transcripts involved in the intrinsic apoptotic pathway was quantified, showing BAX increased after BPA and BPF exposure. BAX is an important pro-apoptotic regulator of apoptosis in the intrinsic pathway and an increase in BAX mRNA in BPA treated cells was detected in murine antral follicles, insulinoma cells and macrophages (10, 29, 30). Despite even fewer studies available on BAX following exposure to BPF, Zhao and colleagues found that murine macrophages exposed to BPF also had an increased expression of BAX with increased apoptosis (54). BAD, another pro-apoptotic protein was also increased in the BPA high dose group at 12 hours and after 48 hours in the BPF high dose group. BAD is a protein that can regulate apoptosis through the binding of anti-apoptotic proteins, such as BCL-2, where it can dimerize with BCL-2, displacing BAX, allowing it to increase mitochondrial membrane permeability, thereby mediating the effects of apoptosis (55). Zhao and colleagues found an increase in BAD expression following BPF exposure, while Huang and colleagues found that same increase following BPA exposure (30, 54) Lastly, pro-apoptotic caspase-9 was quantified, as it is an initiator caspase that activates the executioner caspases 3,6 and 7, which leads to cell death (56). Once caspase-9 is activated, the mitochondrial outer membrane permeabilizes, which is considered the point of no return leading to inevitable cell death (57). In the present study, it was found that caspase-9 mRNA was increased in the BPA high dose at 12 hour and in the BPF high dose at 48 hour. Similar upregulations in expression were also found in ovarian cells, murine sperm, and the ovaries of mice offspring treated with BPA (58–60). When it comes to BPF, caspase-9 mRNA was increased in zebrafish larvae and in macrophages (54, 61). Caspase-9 is an important component of the intrinsic pathway of apoptosis and the upregulation of this caspase provides us with more evidence that apoptosis induced by BPA and BPF occurs through the intrinsic pathway.

Maintaining a balanced ratio of anti-apoptotic BCL-2 to pro-apoptotic BAX proteins is what allows a cell to remain in a healthy, non-apoptotic state. In a healthy cell, BAX remains in the cytosol and only minimally binds to the outer mitochondrial membrane (OMM), while BCL-2 ensures that it does not accumulate (62). BAX binds to anti-apoptotic proteins, such as BCL-2, which inhibit BAX’s translocation to the OMM, however, if this ratio favors BAX, apoptosis can occur (62).

Anti-apoptotic BCL-2 mRNA was significantly increased in the BPA high dose group after 12 hours of treatment and in the BPF high dose group at 48 hours. These results mimic the increase in expression seen in the pro-apoptotic BAX, BAD and caspase-9 genes. Although BCL-2 expression should be downregulated as apoptosis is occurring, one can speculate that the cell is trying to compensate for the increase in pro-apoptotic BAX by increasing the anti-apoptotic genes as a means to maintain the BAX/BCL-2 ratio, thereby returning the cell to its normal state to prevent apoptosis. Studies have shown that increased expression of BCL-2 slows down apoptosis and increases the time to cell death (63). Some studies have found a decrease in BCL-2 expression after bisphenol treatment, such as Gu et al. who treated zebrafish with BPF and found their spawn to have downregulated BCL-2 mRNA expression (61). On the other hand, studies have also demonstrated an increase BCL-2 mRNA expression following bisphenol treatment, as detected by Peretz et al. in cultured murine antral follicles (10). This study also found that a higher BCL-2/BAX ratio was not able to protect the follicles from atresia, elucidating that follicles may be up-regulating BCL-2 to prevent atresia and apoptosis, however, with no success as the ratio was still in favor of BAX (10) Another important note is this increase in mRNA could be due to an effect on the protein level as transcript abundance is not always directly proportional to protein abundance (64). Therefore, it is crucial to quantify BAX and BCL-2 at the protein level to determine the plausible effects of these bisphenols.

HSP70 is another anti-apoptotic gene that functions as a chaperone, has cytoprotective properties, and aims to prevent cell death that is triggered by various stimuli (65). It was found that HSP70 mRNA was significantly elevated only in the BPA high dose group. While under stressful or lethal conditions, such as exposure to environmental toxins, the elevation of HSP70 can help cells cope by blocking apoptosis (65). HSP70 can stabilize Akt/PKB that targets apoptotic proteins such as BAD and caspase 9, interfering with their activation (66, 67). HSP70 can also block the translocation of BAX to the OMM, thus preventing mitochondrial outer membrane permeabilization and inhibiting the release of cytochrome C (65). Based on this information, the increase observed after 12 hours of BPA treatment, may be the cells’ attempt to mediate the effects of apoptosis, likely trying to interfere with caspase-9, BAX, or BAD activation. Multiple studies have found an increase in HSP70 expression following BPA exposure in various models, such as salmon kidney cells, Asian paddle crab tissues, and dinoflagellate cells (68–70). Programmed cell death is mediated by caspases, which are proteases that cleave their substrate and activate the caspase cascade (71). While we determined there was an increase in caspase-9 mRNA expression following BPA and BPF exposure, assessing the enzymatic activity of caspase-9 further allowed us to determine the signal transduction pathway of bisphenol-induced apoptosis. In this study, caspase-9 activity was strongly induced by BPA followed by BPF at the high dose at 12 and 48 hours, respectively. Another key observation that indicates BPF may be more potent than BPS and is more similar to BPA in terms of mechanistic actions. Mokra and colleagues found similar increases in caspase-9 activity in human peripheral blood mononuclear cells treated with BPA and BPF at the same concentration of 50 ug/mL (72). In human placental cells, caspase-9 was significantly activated post BPA and BPF treatment with BPS having no effect on caspase activation (33).

Lastly, this study investigated if the changes in BAX and BCL-2 mRNA led to translational changes at the protein level. There was an increased BAX/BCL-2 ratio in the BPA high dose groups at 12 hours with increased and decreased BAX and BCL-2 protein expression, respectively. Interestingly, at the later timepoint, BAX/BCL-2 ratio was decreased in favor of BCL-2 in the BPF low dose group. This may suggest that a lower dose of this analog does induce oxidative damage in the cells which then increase BCL-2 protein levels to combat this damage and prevent apoptosis. This is supported with the lack of apoptotic detection with BPF at this dose through annexin staining. Furthermore, BPF is shown to have a weaker potency than BPA indicating that granulosa cells could be more resistant to the effects of this analog at lower doses (73). A longer timepoint might be needed to see the toxic effect of BPF at this low dose. Overall, these results align with those in the literature showing an increased protein expression of BAX with a decrease in BCL-2 expression following exposure to BPA. This includes human granulosa cells, murine macrophages, and acute myeloid leukemia cells, to name a few (23, 30, 74), supporting that BPA likely induces apoptosis predominantly through the intrinsic pathway by influencing the levels of BAX to BCL-2 proteins present around the mitochondria.

A notable finding of this study is the similarities and differences between BPA and BPF. Both proved to induce changes in granulosa cell morphology, reduce live cell counts, increase most apoptotic transcripts, influence BAX/BCL-2 ratios, activate caspase 9, and induce early apoptosis through annexin positive staining. This is not surprising as these compounds are structurally similar, and studies have reported that BPF shares estrogenic and androgenic activities with BPA (75). In recent years, studies comparing BPA and BPF within the context of female reproduction has rapidly grown. In zebrafish, BPF exposure resulted in reproductive toxicity of the offspring with enrichment of cancer and tumor formation pathways (76) indicating that BPF effects on female reproduction can lead to transgenerational effects. In mammals, BPF disrupted gene expression and promoted trophoblast invasion in endometrial stromal cells (77), further studies found that BPF could regulate steroid hormone receptor function interfering with endometrial cell receptivity and thereby impairs embryonic implantation (78). Within follicular development, a recent study investigated the effects of BPF on bovine theca cells and reported no changes in theca cell viability but did observe disruptions in progesterone production (79). Another study reported similar findings in porcine granulosa cells; unlike BPA, BPF also had no effect on granulosa cell viability, but did disrupt gene expression of key factors for follicular development (80). BPF is also reported to induce oxidative stress in human and bovine granulosa cells (14, 81). BPF has also shown to be correlated with negative effects on male fertility as well. Fatai et al. noted key associations between BPF exposure and reduced semen quality in male wistar rats including decreased sperm motility and viability (82). Lastly, BPF was shown to trigger apoptosis in human endometrial cells with induced caspase activation (83). Overall, the current literature on BPA analogues in the context of female reproduction is limited with most investigations done on other reproductive tissues/cells besides granulosa cells and those that do use granulosa cell models have focused on pathways involved in steroid hormone production, follicular development, and oxidative stress with limited research on apoptotic pathways. This speaks to the novelty of the presented research and the need to expand our knowledge on the effects of these widespread analogues.

Although they shared the same effects, a crucial observation was the delayed response for BPF to induce the same changes as BPA at the same dose; BPA significantly disrupted the granulosa cells after 12 hours of exposure, while BPF presented similar disruptions after 48 hours. This speaks to the degree of potency for the different types of bisphenols. Kinetic studies have determined that out of three bisphenols tested, BPA is the most potent as it is more lipophilic with a higher dissociation constant while BPS is the least lipophilic with the lowest dissociation constant and BPF is an intermediate between the two (73). This indicates that BPA and BPF will accumulate in tissues to a greater degree than BPS and once metabolized, they can easily dissociate into their toxic free forms (84).

In our current study, BPS did not induce bovine granulosa cell apoptosis. This is evident as BPS did not display an effect on cell counts, cause any morphological changes in the cells or induce apoptosis as measured through annexin staining. This study found no indicators that the intrinsic pathway of apoptosis was activated. Studies in literature have shown BPS may work through an alternative mechanism to induce apoptosis in other cell/tissue types (33). In human placental cells, Fouyet et al. found that BPA induced intrinsic apoptosis as it activated caspase-9, BPF induced both intrinsic and extrinsic apoptosis through activation of caspase-8 and -9, and BPS induced apoptosis through a caspase-independent mechanism as it did not activate any caspases (33). On the other hand, Vinas and Watson found that BPS activated caspase-8 from 4 hours onwards, and weakly activated caspase-9 only at 24 hours in rat pituitary cells (85). This led authors to conclude that the primary mechanism through which BPS acts to induce apoptosis is the extrinsic pathway, and that the later activation of caspase-9 was likely due to pathway crossover through the BCL-2 interacting protein (BID) in the caspase-8 pathway that led to release of cytochrome c and activation of caspase-9 (85). In peripheral blood mononuclear cells, Mokra et al. showed that BPS increased the activity of caspase-8, while also activated caspase-9, concluding, similarly to Vinas and Watson, that BPS activates the extrinsic pathway and subsequently the intrinsic pathway through BID (72, 85) Another possible explanation is that the dosage at which the cells were treated did not induce effects, however, a higher dosage may. The high dose used in our study is the LOAEL established for BPA, which is 50 mg/kg bw/day. There is limited toxicological data when it comes to BPS in humans and animals. In adult rats, a LOAEL for parental toxicity was established to be 60 mg/kg bw/day, while the LOAEL for reproductive toxicity was set to 300 mg/kg bw/day (86, 87). Another study established a different NOAEL for fertility and reproductive performance in rats to be 180 mg/kg body weight/day, which is three times higher than the previous study (88). Both studies found the LOAEL for BPS to be much greater than the doses used in our study. This suggests that perhaps a higher dosage was required to see a difference in cell viability for BPS. In human placental cells, Fouyet et al. found no loss in cell viability after 72 hours with BPS treatment up to 100 μM, which is a lower dose than this study used (33). These findings, in conjunction with current literature, suggest that BPS may not be as toxic as BPA and BPF.

On the other hand, there is also evidence that BPS may have a nonmonotonic dose response relationship, whereby lower doses induce more potent effects than higher doses (89). For example, pre-pubertal mice treated with 10 μg/kg body/weight day (0.01 mg/kg), a dose lower than this study (0.05 mg/kg) resulted in decreased *in vitro* fertilization rates and an increased number of abnormal oocytes (90). In male mice, sperm motility, protein methylation and acetylation were altered by a BPS dose of 0.001 μg/kg body weight/day, however not at higher doses (91). Further studies have found evidence of this nonmonotonic dose relationship which suggests that perhaps the little to no effect seen in this study was due to the lowest dose being higher than minute doses reported to produce an effect. This evidence, coupled with the evidence above regarding the LOAEL, could possibly explain why both BPS doses in this study did not affect cell viability. The findings in this study along with findings in literature present the challenges of establishing strict parameters and standardized LOAELs for BPA and its analogues considering bisphenol mediated responses can be species-dependent and tissue/cell-dependent. Furthermore, non-monotonic dose responses present additional obstacles for determining what is a ‘safe’ level of bisphenol exposure. Growing research on various systems utilizing more than one exposure dose, as done in this study, will add to the body of knowledge needed to begin establishing appropriate LOAEL doses for different types of bisphenols.

## Conclusion

In conclusion, this study increases our knowledge of the effects of BPA and its analogs, BPS and BPF, in bovine granulosa cells. The findings of this report strongly suggest that BPF had the most profound effects of the two BPA analogues tested. Our results support that BPA and BPF decrease cell viability through the induction of apoptosis. Further, the results of our study increase our understanding of the mechanism through which bisphenols act to induce apoptosis. BPA and BPF induced changes in the mRNA and protein expression and activity of key components of the intrinsic pathway of apoptosis, suggesting that this may be the predominant mechanism used by both. However, the effects of BPS remain inconclusive, and so does the mechanism through which it may act. Overall, these findings present novel data that suggests the BPA’s analogue, BPF, is able to induce apoptosis in bovine granulosa cells, thus contributing to disrupted granulosa cell function.

## Data availability statement

The raw data supporting the conclusions of this article will be made available by the authors, without undue reservation.

## Author contributions

Conceptualization, Methodology: EK, RS, and LF. Supervision, Methodology: LF. Data curation, Writing-Original draft preparation: EK. Experiments *in vitro*: EK. Conceptualization, Design: EK, RS, and LF. Writing, Editing: EK, RS, and LF. All authors contributed to the article and approved the submitted version.

## Funding

This work was supported by the Natural Sciences and Engineering Research Council of Canada (NSERC) [Grant #401511, 2019] and the Ontario Veterinary College (OVC) Scholarship in the Department of Biomedical Sciences at the University of Guelph.

## Acknowledgments

The authors wish to thank Dr. W. Allan King for the stimulating discussions, Dr. Monica Antenos, Elizabeth St. John, and Allison MacKay for excellent technical assistance and all the members of the Reproductive Health and Biotechnology Laboratory (University of Guelph) for continuous support. A huge thank you to Cargill Meat Solutions (Guelph, ON) and Highland Packers (Stoney Creek, ON) that provided the ovaries necessary for the retrieval of granulosa cells.

## Conflict of interest

The authors declare that the research was conducted in the absence of any commercial or financial relationships that could be construed as a potential conflict of interest.

## Publisher’s note

All claims expressed in this article are solely those of the authors and do not necessarily represent those of their affiliated organizations, or those of the publisher, the editors and the reviewers. Any product that may be evaluated in this article, or claim that may be made by its manufacturer, is not guaranteed or endorsed by the publisher.
