# Supplementary figures and images for: Bisphenols A and F, but not S, induce apoptosis in bovine granulosa cells via the intrinsic mitochondrial pathway
Source: Front Endocrinol (Lausanne). 2022 Oct 28;13:1028438. doi: 10.3389/fendo.2022.1028438 (PMC9650025; doi:10.3389/fendo.2022.1028438)

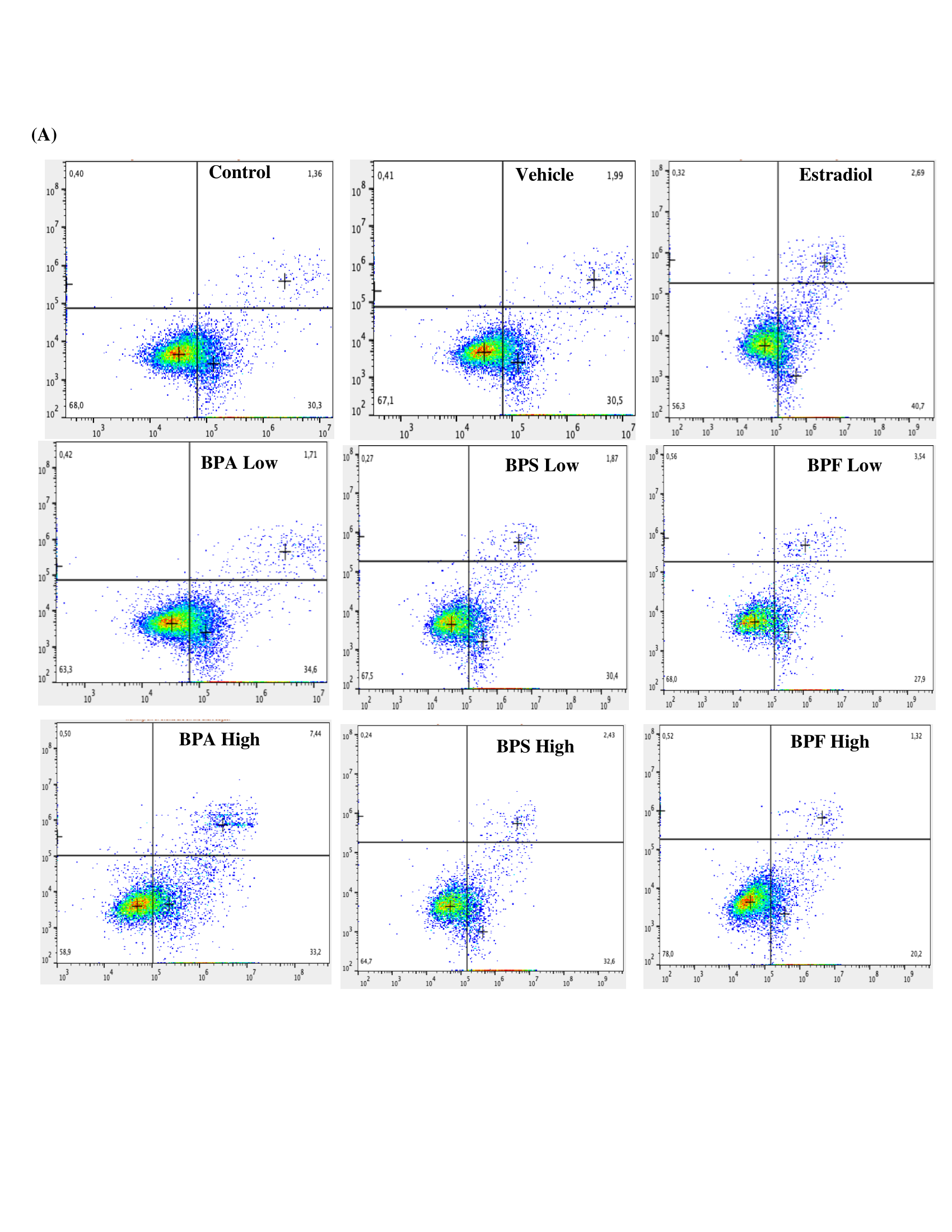

Supplement: Supplementary file 1 [file Image_1.tiff]
